# Elevation of endocannabinoids in the brain by synthetic cannabinoid JWH-018: mechanism and effect on learning and memory

**DOI:** 10.1038/s41598-019-45969-4

**Published:** 2019-07-03

**Authors:** Ren-shi Li, Ryo Fukumori, Tomoki Takeda, Yingxia Song, Satoshi Morimoto, Ruri Kikura-Hanajiri, Taku Yamaguchi, Kazuhito Watanabe, Kousuke Aritake, Yoshitaka Tanaka, Hideyuki Yamada, Tsuneyuki Yamamoto, Yuji Ishii

**Affiliations:** 1 Laboratory of Molecular Life Sciences, Graduate School of Pharmaceutical Sciences, Kyushu University, 3–1–1 Maidashi, Higashi-ku, Fukuoka 812–8582 Japan; 2 Research Department of Pharmacognosy, China Pharmaceutical University, Nanjing, 211198 People’s Republic of China; 3 Department of Pharmacotherapeutics and Neuropsychopharmacology, Faculty of Pharmaceutical Sciences, Nagasaki International University, Sasebo, Nagasaki Japan; 4 Division of Pharmacognosy, Graduate School of Pharmaceutical Sciences, Kyushu University, 3-1-1 Maidashi, Higashi-ku, Fukuoka 812-8582 Japan; 5 Division of Pharmacognosy, Phytochemistry and Narcotics, National Institute of Health Sciences (NIHS), 3-25-26 Tonomachi, Kawasaki-ku, Kawasaki-city, Kanagawa 210-9501 Japan; 6 Daiichi University of Pharmacy, 22-1 Tamagawa-cho, Minami-ku Fukuoka, 815-8511 Japan; 7 Division of Pharmaceutical Cell Biology, Graduate School of Pharmaceutical Sciences, Kyushu University, 3-1-1 Maidashi, Higashi-ku, Fukuoka 812-8582 Japan

**Keywords:** Metabolomics, Short-term memory, Diagnostic markers

## Abstract

The impairment of learning and memory is a well-documented effect of both natural and synthetic cannabinoids. In the present study, we aimed to investigate the effect of acute administration of JWH-018, a synthetic cannabinoid, on the hippocampal metabolome to assess biochemical changes *in vivo*. JWH-018 elevated levels of the endocannabinoids, anandamide (AEA) and 2-arachidonoylglycerol (2-AG). The increase of endocannabinoid levels in response to JWH-018 could be inhibited by co-administration of AM251, a CB1 receptor antagonist. Biochemical analyses revealed that this was the result of suppression of two hydrolases involved in endocannabinoid degradation (fatty acid amide hydrolase [FAAH] and monoacylglycerol lipase [MAGL]). Additionally, we showed that JWH-018 causes a reduction in the levels of brain-derived neurotrophic factor (BDNF), which is known to modulate synaptic plasticity and adaptive processes underlying learning and memory. The decrease of BDNF following JWH-018 treatment was also rescued by co-administration of AM251. As both endocannabinoids and BDNF have been shown to modulate learning and memory in the hippocampus, the alteration of their levels in response to JWH-018 may explain the contribution of synthetic cannabinoids to impairment of memory.

## Introduction

Synthetic cannabinoids have recently been considered as a popular alternative to Δ^9^-tetrahydrocannabinol (Δ^9^-THC, the principle psychoactive ingredient of *Cannabis sativa*)^1^, due to an increasing demand of this product and the constant variants in their analog structural forms. Naphthalen-1-yl-(1-pentylindol-3-yl)methanone (JWH-018) is a synthetic cannabinoid illegally marketed as ‘Spice’ and ‘herbal blend’ with psychoactive effects greater than Δ^9^-THC^2,3^. The impairment of learning and memory is one of the major cognitive effects caused by consumption of both natural and synthetic cannabinoids^4–8^.

The effects of synthetic cannabinoids are mediated through the cannabinoid receptor type 1 (CB1 receptor)^9^, which is predominately expressed in the hippocampus, a brain region essential for learning and short-term memory^10–13^. Therefore, the impairment on learning and memory elicited by JWH-018 has so far been attributed to effects on this specific brain region. Currently, inferring specific health risks associated with the use of synthetic cannabinoids is challenging as limited toxic and pharmacological information is available, and the exact cellular mechanisms underlying memory and learning impairment elicited by substances such as JWH-018 are unclear. Metabolomics, the comprehensive study of global metabolites, is a highly sensitive and powerful tool for systemic toxicological approach^14,15^, and may provide comprehensive information on the dynamic changes induced by JWH-018. Therefore, we aimed to investigate the effects of JWH-018 on the hippocampal metabolome of mice to elucidate the potential mechanism of the impairment in learning and memory.

Notably, in this study, we discovered that JWH-018 increases the content of anandamide (AEA) and 2-arachidonoylglycerol (2-AG) in the hippocampus of mice by metabolomics for the first time. Those are both endocannabinoids as well^16–18^. It is known that endocannabinoids exhibit a profound impact on neuronal communication, including learning and memory through the activation of CB1 receptors to suppress neurotransmission^6,19^. Furthermore, several studies have shown that the endocannabinoid system plays an important role in learning and memory in the hippocampus^20,21^. Thus, quantitation of these endocannabinoids from hippocampal tissues was essential for elucidation of cannabinoid-mediated processes *in vivo*. Usually, endocannabinoids are rapidly degraded, therefore we elucidated the biosynthetic and metabolic pathways for AEA and 2-AG. In addition, brain-derived neurotrophic factor (BDNF) is known to be involved in hippocampus-dependent learning and memory^22–24^. It is a potent synaptic modulator expressed throughout the hippocampus, and has been shown to play a critical role in many behavioral processes^25,26^. Therefore, we examined whether JWH-018 affects the level of BDNF in the hippocampus.

In our study, we evaluated memory impairment using behavioral tests, and quantified endocannabinoids and BDNF expression after the administration of JWH-018 in mice. Our results may provide a better understanding of the mechanism of the toxicity elicited by JWH-018.

## Results

### Behavioral studies

**Figure 1:**
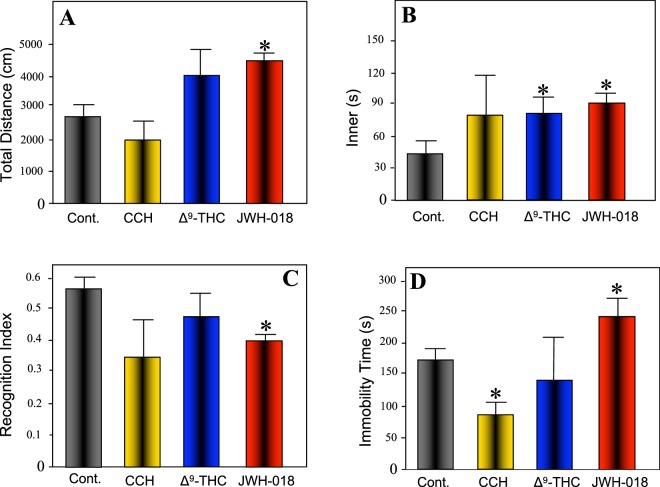
Effect of exposure to CCH, Δ^9^-THC and JWH-018 on locomotor activity (**A**), anxiety-like behavior (**B**), and learning memory (**C**,**D**) in male C57BL/6J mice. (**A**,**B**) Locomotor activity and effects on anxiety-like behavior were assessed using the open field test. (**C**) Recognition memory was measured using the novel object recognition test. (**D**) Antidepressant-like activity was determined by the tail suspension test. All drug-treated groups were compared with the respective vehicle-treated groups. Each bar represents the mean ± SEM of 5 mice for each treatment. *p < 0.05 indicates a significant difference compared to controls.

In order to evaluate the effects of acute exposure to JWH-018, mice were injected once with either a vehicle or JWH-018 (1 mg/kg, i.p.). We commonly observed induction of abnormal symptoms, such as rotational and seizure-like behavior, by JWH-018 at least one hour after administration. Behavioral analyses were conducted two hours after administration of JWH-018. Locomotor and anxiety-like behavior were evaluated in the open-field test, and novel object recognition (NOR) as well as tail suspension tests were conducted to compare amnesic effects of JWH-018 to those of cannabicyclohexanol (CCH) and Δ^9^-THC. The results of the open-field test suggested that JWH-018 increases locomotor exploration while decreasing anxiety-like behavior in a novel environment (Fig. 1A,B). In addition, NOR test revealed a decrease in the recognition index of the JWH-018 group when compared with the control group (Fig. 1C). On the basis of this observation, it was strongly suggested that JWH-018 produces an impairment of memory retention in mice, which is in concordance with previous reports of memory impairments resulting from acute injections of JWH-018^27^. Along these lines, tail suspension tests revealed a higher immobile time for the JWH-018 group, whereas it was shortened in the CCH group compared with vehicle-injected controls (Fig. 1D). These results could help to identify a behavioral pharmacological marker for synthetic cannabinoid abuse in the future.

### Change in the hippocampal metabolome following JWH-018 in mice

**Figure 2:**
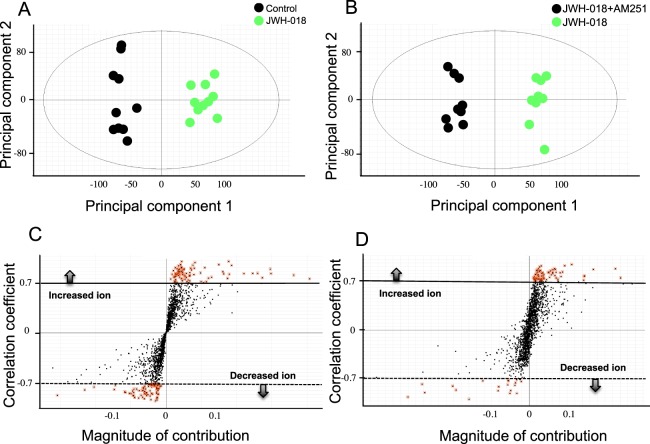
Metabolomic profiling of the JWH-018-treated group compared to control or co-administration of AM251 using UPLC-TOF/MS. Panels A and C represent the OPLS and S-plot mode of the control versus JWH-018 group. Panels Band D represent the OPLS and S-plot mode of the JWH-018 group versus co-administration of AM251 group. Fragment ions with correlation coefficients of more than +0.7 and less than −0.7 are shown as red dots (panels C and D). 10 animals were used per group.

Hippocampal extracts were subjected to metabolomic analysis using UPLC-TOF/MS. The principal component analyses of the metabolomic data obtained from UPLC-TOF/MS in positive-ion modes (Fig. 2A,B) revealed that the clusters of plots in the two groups were distinctly separated from each other. This observation suggests that the hippocampal metabolome varies markedly between JWH-018 treatment and JWH-018/AM251 co-administration. The S-plot was used to select potential biomarkers and visualize metabolomic changes. The farther red dots were the more likely biomarker candidates because of their high contributions and correlations (Fig. 2C,D).

By aligning mass and retention times of ions to a previous database, we observed that many metabolites were significantly altered, including as amino acids, endocannabinoids, and tricarboxylic acid (TCA)-cycle intermediates (see the Supplemental Table 1 for the detailed conditions). Due to the nature of these observations, some ingredients were further investigated. This reflects the metabolic response of mice to JWH-018 exposure revealing potential markers of metabolomic perturbation. Furthermore, providing sufficient data to foray on the research and exploration of its underlying mechanisms. For example, we observed a reduction of N-acetyl-aspartate (NAA) in the brain following JWH-018 exposure. NAA is typically present at a high concentration in the brain and is a neuron-specific marker for monitoring neuronal damage associated with central nervous system (CNS) diseases although it lacks specificity for any particular disease^28,29^. Therefore, reduction of NAA suggested neuronal damage following JWH-018 exposure. Additionally, glutamic acid, a major excitatory neurotransmitter in the brain, was increased in the JWH-018-treated group compared to the control group. Furthermore, administration of JWH-018 resulted in increases of phenylalanine and tyrosine levels, and succinic acid, which is an intermediate of the TCA-cycle indicating the influence of the TCA cycle for energy metabolism and supply. Although we did not address the functional meaning of these significantly varied metabolites in depth in this study, understanding the contribution of these metabolite alterations may potentially aid clarifying the effects of synthetic cannabinoids on the CNS in the future.

Importantly, we observed for the first time that JWH-018 treatment resulted in a significant elevation of AEA and 2-AG levels, which may indicate alterations in fatty acid metabolism. AEA and 2-AG are endogenous ligands of the CB1 receptor, serving as mediators for the retrograde suppression of synaptic transmission and modulate synaptic plasticity^27,30,31^, which plays a pivotal role in learning and memory. An accumulation of endocannabinoids suppressing synaptic long-term potentiation could elicit an aggravation of memory impairment. Therefore, it was important to confirm whether JWH-018 induces the accumulation of endocannabinoids. Furthermore, application of a specific CB1 receptor antagonist could help to elucidate the mechanism underlying an increase in endocannabinoids by JWH-018.

### Determination of endocannabinoids (AEA and 2-AG) concentrations in the hippocampus after JWH-018 treatment

The content of hippocampal endocannabinoids was determined using methods reported previously^32^ with slight modifications. Identification and quantification of AEA and 2-AG were performed using an UPLC-TOF/MS (LCT-Premier XE, Waters, Milford, MA) with TargetLynx^XS^ v4.1 software (Waters Co., Milford, MA). A five point calibration curve with concentration ranges of 0.01–1 μM (0.01, 0.05, 0.1, 0.5, 1) of AEA*-d*_4_ and 3.125–50 μM (3.125, 6.25, 12.5, 25,50) of 2-AG-*d*_8_, and blanks with and without the internal standards were prepared. A linear regression of the ratio of the peak area counts of AEA, 2-AG to their corresponding internal standards (anandamide-*d*_4_ and 2-AG-*d*_8_) vs. concentration was used to construct the calibration curves (Supplemental Table 2).

**Figure 3:**
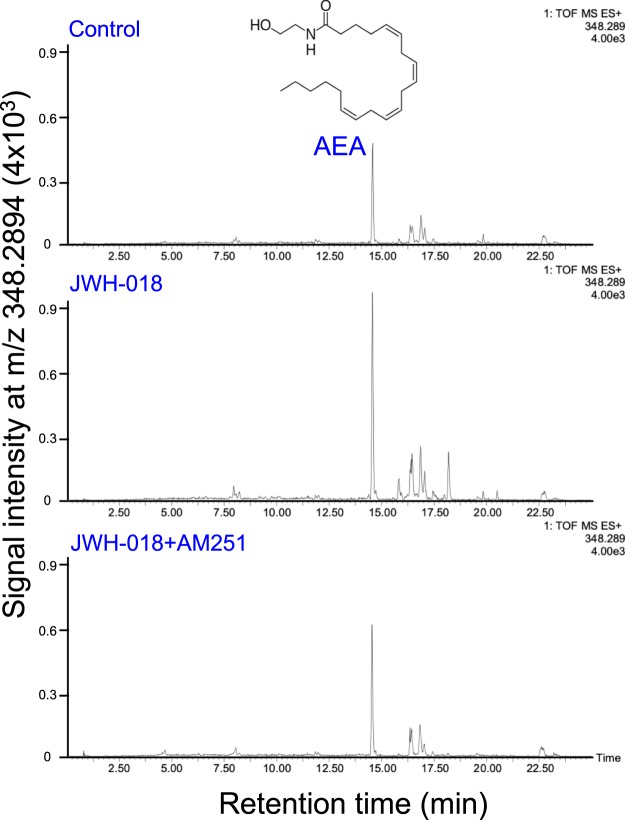
Selected ion monitoring of AEA in the hippocampus of C57BL/6J mouse. Selected ion monitoring at m/z 348.2895 for the determination of the hippocampus content of AEA. The retention time for AEA was 14.54 min. (**A**) Control hippocampus extract; (**B**) hippocampus extract from JWH-018-treated mice; (**C**) hippocampus extract from JWH-018- and AM251-treated mice. One of the representative data of each group is shown.

**Figure 4:**
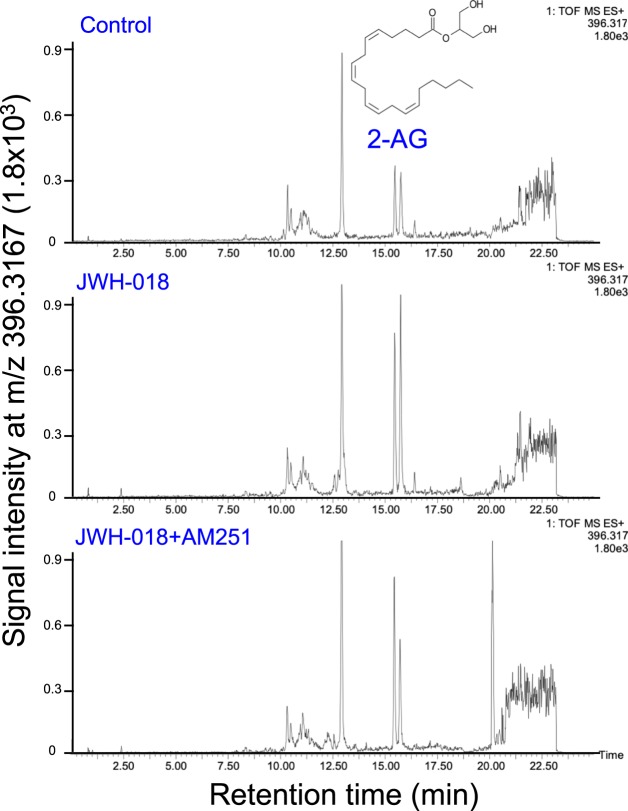
Selected ion monitoring of 2-AG in the hippocampus of C57BL/6J mouse. Selected ion monitoring at m/z 396.3117 for the determination of the hippocampus content of 2-AG. As 2-AG undergoes isomerization to 1-AG arising from acyl group migration during sample workup, the peak areas of both isomers were combined for 2-AG quantitation. 2-AG and 1(3)-AG (retention times 15.44 and 15.74 min, respectively). (**A**) Control hippocampus extract; (**B**) hippocampus extract from JWH-018-treated mice; (**C**) hippocampus extract from JWH-018- and AM251-treated mice. One of the representative data of each group is shown.

**Figure 5:**
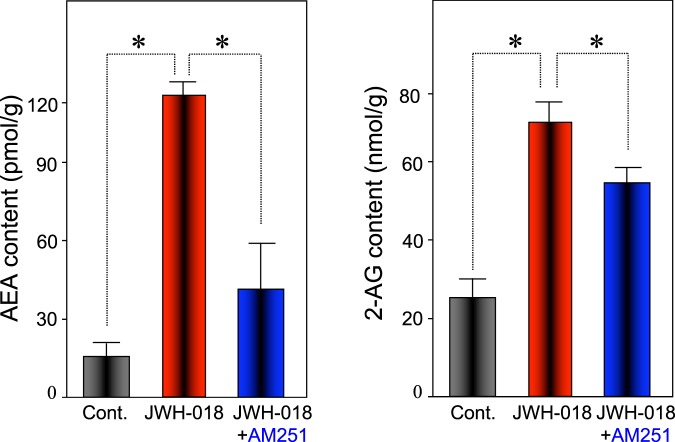
JWH-018 elicits the accumulation of AEA and 2-AG in the hippocampus of C57BL/6J mice. The content of AEA and 2-AG in hippocampus of male mice was determined using of UPLC-TOF/MS. The bars represent the means ± SEM. of 5 mice. *p < 0.05 indicates a significant difference compared to controls.

As shown in Figs 3–5, UPLC-TOF/MS analysis was performed in a selected ion-monitoring mode. The mean AEA and 2-AG tissue levels in the control group were determined to be 11.6 pmol/g and 26.5 nmol/g of tissue, respectively, which is in concordance with previous reports^33,34^. Furthermore, this measurement confirmed that JWH-018 caused a 10.6- and 2.7-fold increase of these components in the hippocampus, respectively. This elevation was blocked by co-administration of AM251. Taken together, our results demonstrate that JWH-018 facilitates endocannabinoids accumulation in a CB1 receptor-dependent fashion in brain hippocampus.

### Mechanism underlying the JWH-018-elicited increase AEA and 2-AG in the hippocampus

**Figure 6:**
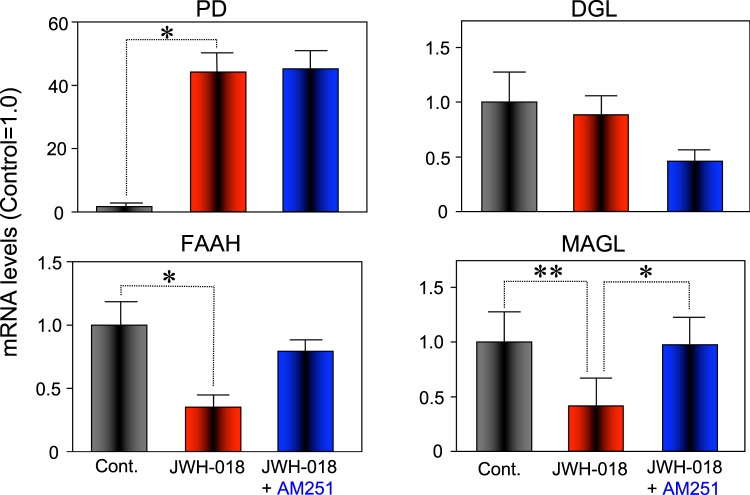
Effect of JWH-018 on the mRNA expression of AEA and 2-AG synthesizing and degrading enzymes, respectively, in the hippocampus of male mice. Mice were exposed to either JWH-018 (1 mg/kg) or vehicle, and their hippocampi were collected 8 h after treatment. The relative levels of mRNAs indicated were analyzed by real-time RT-PCR and normalized to β-actin mRNA. Each bar represents the means ± SEM of 4–6 animals. *p < 0.05 or **p < 0.01 indicate significant difference compared to controls.

Total protein and RNA extraction were carried out after behavioral tests. Mice were euthanized and hippocampi were rapidly collected, snap-frozen in liquid nitrogen, and stored at −80 °C until use. To investigate how JWH-018 treatment elicits accumulation of endocannabinoids in the hippocampus, we examined the expression of mRNAs coding for endocannabinoid synthesizing and metabolizing enzymes. Biosynthesis of AEA is primarily achieved by the enzyme N-acyl-phosphatidylethanolamines-hydrolyzing phospholipase D (PD)^35^. Fatty acid amide hydrolase (FAAH) is an intracellular membrane-bound enzyme that degrades fatty acid amides and it is responsible for inactivation of AEA by catalyzing its breakdown to arachidonic acid (AA) and ethanolamine^36,37^. The primary synthetic and metabolic enzymes for 2-AG have been identified as diacylglycerol lipase (DGL) and monoacylglycerol lipase (MAGL), respectively^38,39^. JWH-018 treatment decreased the mRNA level of FAAH and MAGL. Conversely, co-treatment with AM251 completely restored the mRNA levels of these enzymes (Fig. 6).

### Alteration of BDNF expression in the hippocampus by JWH-018

**Figure 7:**
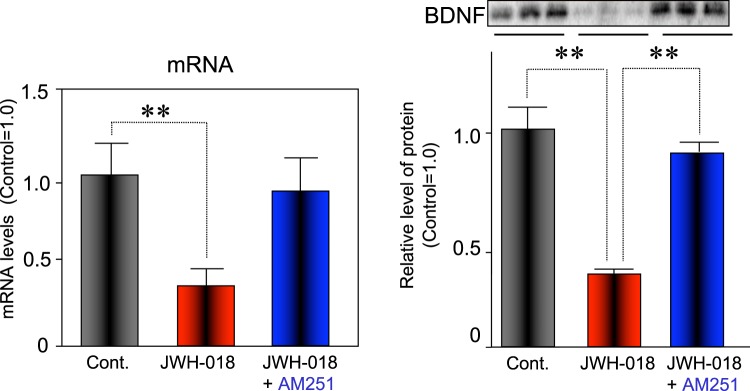
Effect of administration of JWH-018 on BDNF mRNA and protein levels in the hippocampus. The bars represent the means ± SEM of 3 mice. **p < 0.01 indicates significant difference compared to controls.

By evaluating the mRNA and protein levels of BDNF, a marker of synaptic plasticity in the hippocampus, we aimed to investigate molecular changes that can be linked to behavioral effects caused by JWH-018. BDNF is known to modulate neuroplasticity and adaptive processes underlying learning and memory^40^. BDNF was significantly decreased following exposure to JWH-018 both at mRNA and protein levels (Fig. 7). This effect was prevented by co-administration of AM251.

## Discussion

In this study, we investigated the effect of administration of the synthetic cannabinoid JWH-018 on the hippocampal metabolome. We discovered that the levels of AEA and 2-AG were significantly elevated in the hippocampus of mice treated with 1 mg/kg of JWH-018 compared with control groups (Fig. 5). This increase was inhibited by co-administration of AM251, a CB1 receptor antagonist. Further analyses revealed that the elevation of endocannabinoids were the result of the suppression of two hydrolases (FAAH and MAGL). The fold change of AEA was greater than that of 2-AG (Fig. 5). As the constitutive levels of AEA are far lower than of 2-AG (Fig. 5), it is possible that AEA is more sensitive to alterations in the expression of metabolic enzymes than 2-AG. Additionally, our behavioral studies were in concordance with previous reports of memory impairments resulting from acute injections of JWH-018^41^. Similarly, electrophysiological and neurochemical data previously demonstrated that synthetic cannabinoids impair cognitive function by interfering with hippocampal synaptic transmission and other mechanisms regulating memory^11,41^. Administration of either AEA or 2-AG inhibits long-term potentiation of hippocampal field excitatory postsynaptic potential (EPSP)^42,43^. Recent studies carried out with metabolically stabilized AEA have revealed inhibitory effects on learning and memory^44,45^. Therefore, the accumulation of endocannabinoids may be one of the factors involved in the impairment of cognitive functions elicited by JWH-018.

Furthermore, our data suggest that JWH-018 impairs BNDF expression (Fig. 7). BDNF is an important modulator of excitatory and inhibitory synaptic transmission, and has been described as capable of mediating several cellular events, including neuronal differentiation and growth, synapse formation and plasticity, as well as higher cognitive functions^46,47^. Therefore, memory impairments elicited by JWH-018 may also be due to decreases in BDNF.

‘Spice/K2’ is illegally marketed as a natural herbal blend but contains at least one highly potent synthetic CB1 receptor agonist, which likely accounts for the cognitive deficits psychoactive effects produced by consumption^11^. So far, the mechanisms through which the synthetic cannabinoid JWH-018 found in ‘Spice’ and ‘K2’ impairs memory have remained to be clarified. The present study suggests that JWH-018 affects cellular events and hippocampal function by CB1-mediated signaling, resulting in potentiation of endocannabinoids and a decrease of BDNF levels. However, further studies are necessary to elucidate more details of the underlying molecular mechanisms. The establishment of a method for detecting and quantifying endocannabinoids in the hippocampus as described in this study will be critical to assist further investigation of the complex effects of synthetic cannabinoids on behavior. Similarly, it could contribute to the forensic investigation of post-mortem cases or impaired driving when synthetic cannabinoid use is suspected.

To the best of our knowledge, this is the first report to evaluate the metabolomic changes in mice after JWH-018 exposure. Consequently, marked changes in some principal metabolites were also detected. It is reasonable to suppose that the identified metabolites and their metabolic pathways relate further metabolic changes after JWH-018 exposure. Generally, there are significant differences in some aspects of mouse and human metabolism. Keeping this limitation in consideration, however, this basic study using mice is still informative as there are similarities in many aspects between mice and humans^48^, and information generated from animal studies will doubtlessly shed light on mechanism of synthetic cannabinoids. As it is currently difficult to gain approval for the investigation of synthetic cannabinoid effects in humans, animal studies will provide meaningful knowledge for both law enforcement and forensic toxicologists.

We demonstrated that metabolomic methods, based on UPLC-TOF/MS, are an effective tool for exploring biomarkers of JWH-018 abuse. We detected several referential biomarkers candidates altered by JWH-018 treatment. To assess whether these putative biomarkers are specific for acute toxicity of JWH-018, further metabolomic studies on chronic application of JWH-018 and other drugs are needed. Cannabinoids have therapeutic potential in several diseases including cancer, pain, and stress^49^. However, clinical use is limited due to adverse effects such as memory impairment^3^. We observed differences between JWH-018 and CCH in the tail-suspension test (Fig. 1), with a higher immobile time following JWH-018 administration. Higher immobile time usually reflects a depressive-like status, but has also been demonstrated to indicate some types of memory impairment^40^. This revealed that behavioral effects can be distinguished between the two synthetic cannabinoids. Moreover, these findings suggest that CB1 receptor antagonists could have a therapeutic benefit for cognitive dysfunction in JWH-018 abusers. In turn, combining metabolomics with behavioral tests could be employed to screen for synthetic cannabinoids with safer characteristics in the future. Therefore, this study also provides invaluable information to develop safer therapeutics against several brain disorders.

## Conclusion

In this study, hippocampal tissue of mice was analyzed to investigate the effects of JWH-018 administration on the metabolic profile. By adopting metabolomics for a systemic toxicological approach, we were able to provide comprehensive information on the dynamics of JWH-018-induced toxicity. Our results indicate that memory impairment following JWH-018 administration may be explained by the elevation of endocannabinoids and the suppression of BDNF in the hippocampus. These results not only provide clarification of the underlying molecular mechanisms of memory impairment caused by JWH-018, but also put forward novel biomarker candidates to predict the abuse of synthetic cannabinoids in forensic toxicology. Brain samples are usually available only in postmortem cases, therefore establishment of practical methods for the determination of elevated endocannabinoids, in blood and urine samples, due to synthetic cannabinoid abuse is future perspective for a non-invasive technique. As there is a vast number of chemicals categorized as synthetic cannabinoid, a common bio-marker could help to identify abuse without the need to directly determine of the exact drug of abuse. Thus, the results of our study provide information on the underlying molecular mechanisms of memory impairment by JWH-018, and may help to better understand the potential risks of synthetic cannabinoids in the future.

## Materials and Methods

### Chemicals and reagents

JWH-018 and CCH were provided from the National Institute of Health Sciences of Japan. 1-(2,4-Dichlorophenyl)-5-(4-iodophenyl)-4-methyl-N-(piperidin-1-yl)-1H-pyrazole-3-carboxamide (AM251), (5Z,8Z,11Z,14Z)-N-(2-hydroxy-[1,1,2,2-D4]ethyl)icosa-5,8,11,14-tetraenamide (AEA-*d*_4_), and 5Z,8Z,11Z,14Z-eicosatetraenoic-5,6,8,9,11,12,14,15-d8 acid 2-glyceryl ester (2-AG-*d*_8_) were purchased from Abcam (Cambridge, UK). Δ^9^-THC was prepared from Cannabis by Professor S. Morimoto. All other reagents were of the highest grade commercially available. All working solutions were stored at −80 °C until use.

### Animal experiment and sample collection

All procedures for the animal experiments in this study were reviewed and approved by the Ethics Committee of Animal Experiments, Kyushu University. All methods were performed in accordance with the relevant guidelines and regulations. Every effort was made to minimize the number of animals used and their suffering. C57BL/6J-Bom mice were purchased at the age of seven weeks. Animals were housed in a cage for one week after their arrival, and were provided with food and water *ad libitum*. The rearing room was controlled on a 12 h light-dark cycle (lights off at 7:00 pm), and the room temperature was maintained at 22 °C. We divided animals in three groups for investigation: JWH-018 treatment, co-treatment with JWH-018 and AM251, and control (vehicle) treatment. In the present study, JWH-018 dose was chosen based on previous studies^41,50^. JWH-018 (1.0 mg/kg injected group) was initially dissolved in absolute ethanol (final concentration was 2%) and Tween-80 (2%) and brought to the final volume with saline (0.9% NaCl). AM251 (1.0 mg/kg) was administered 30 min prior to JWH-018 injection in the co-administration group. The vehicle solution contained 2% ethanol, 2% Tween-80, and saline. Similarly, ∆^9^-THC (3.0 mg/kg) and CCH (1.0 mg/kg) were prepared. Their dose were chosen based on previous studies^51,52^. All drugs were administered intraperitoneally (i.p.), and injected at a volume of 0.1 ml per 10 g of body weight. Behavioral analyses occurred after administration of appropriate drug or vehicle treatment. After the behavioral tests, all the mice were sacrificed for brain sampling collection. The whole brain was placed in liquid nitrogen immediately and frozen at −80 °C until biochemical analysis, along with metabolomics, real-time polymerase chain reaction (RT-PCR), and western blotting.

### Behavioral tests

#### Open-Field Test

Spontaneous locomotor activity was measured in the open field test. Mice were first habituated to an open-field box (60 cm × 60 cm) with black vertical walls and a white floor for 5 min, 2 h after JWH-018 administration. The test animals were allowed to explore the box freely and their behavior (total distance traveled, time spent in the center area of the open field) was recorded for 5 min using the EthoVision XT software (Noldus, Wageningen, Netherlands).

#### Novel Object Recognition Test

The Novel Object Recognition (NOR) test was chosen as it does not involve the retention of a rule and it is entirely based on the spontaneous exploratory behavior of rodents towards objects, therefore representing a working memory test^53^. This test was also performed in an open-field box. In the training trial, each mouse was placed in the open field and allowed to explore two identical objects for 5 min. The test trial was performed 1 h after the training trial. One familiar object and one novel object were placed in the same location as in the training trial. The time spent exploring each object and the total amount of time spent exploring both objects were recorded. The novel object preference was quantified as Recognition Index (RI) calculated as: (novel B/(novel B + familiar A). Using this metric, scores more than 50% values reflect preference for the novel object (good recognition memory) while less than 50% values reflect preference for the novel object (impairment of recognition memory).

#### Tail Suspension Test

The Tail Suspension (TS) test was performed as previously reported^54^. The posture of immobility in the mouse was originally coined ‘behavioral despair’, largely based on the assumption that the animals have ‘given up hope of escaping’. In the present study, mice were suspended 40 cm above the floor by an adhesive tape placed approximately 1 cm from the tip of the tail. Immobility time was recorded during a 5 min period.

## Biochemical Testing

### Metabolomics

The brain was homogenized in four volumes of cold MeOH/CH_3_CN/H_2_O (2:2:1, v/v), vortexed for 30 s and incubated in liquid nitrogen for 1 min. Samples were allowed to thaw and subsequently sonicated for 10 min. Sample lysis in liquid nitrogen combined with sonication was repeated three times. To precipitate proteins, samples were incubated for 1 h at −20 °C, followed by 15 min centrifugation at 13,000 rpm and 4 °C. The resulting supernatant was removed and evaporated to dryness. The dry extracts were then reconstituted in 200 μL CH_3_CN/H_2_O (1:1, v/v). A portion (10 μL) of these samples was subjected to UPLC-TOF/MS (LCT-Premier XE; Waters, Milford, MA) for metabolomic analysis. The operating conditions for UPLC and TOF/MS, and the procedures for multivariate analysis were selected according to the methods described previously^55^. Chromatographic separation was performed on a BEH column (100 × 2.1 mm, 1.7μm i.d.; Waters Corporation, Milford, MA) using a Waters’ ACQUITY UPLC System (Waters Corporation). The duration of the gradient program of 0.1% aqueous formic acid (solvent A) and acetonitrile (solvent B) was 0–2 min, 0.1% B; −6 min, 0.1–25% B; 6–10 min, 25–80% B; 10–12 min, 80–90% B; 12–21 min, 0–99.9% B; 21–23 min, 99.9% B; 23–24 min, 0.1% B; 24–26 min, 0.1% B. The flow rate was 0.3 ml/min.

For MS analysis, scan range was from m/z 50 to 1000 with a scan time of 0.3 s. Source temperature was set to 120 °C with a cone gas flow of 50 L/h. Desolvation gas flow was set to 600 L/h at a temperature of 350 °C. Capillary voltage was set at 2.4 kV for ESI^+^ mode. Mass spectrometry was operated in W optics mode using dynamic range extension. For accurate mass acquisition, a lock mass of leucine-enkephalin was used via a lock spray interface to ensure accuracy during the MS analysis.

### Determination of endocannabinoids in the hippocampus (AEA and 2-AG)

The content of brain endocannabinoids was determined using UPLC-TOF/MS by the methods reported previously with some modifications^32^. Fifty mg of frozen hippocampal tissue were transferred to a borosilicate glass tube containing 1 mL ice-cold acetonitrile spiked with 10 μL of internal standards mixture (1 pmol anandamide-*d*_4_ and 2 nmol 2-AG-*d*_8_). The hippocampus was homogenized manually with a glass pestle, followed by sonication in an ice-water bath for 30 min. The homogenates were kept at −20 °C overnight to precipitate proteins. Samples were then centrifuged at 3000 rpm for 3 min. The supernatant was removed with a glass pipette to a new tube and the process repeated one additional time. The supernatant was dried under a stream of N_2_ gas, resuspended in 300 μL methanol, then evaporated again. The residue obtained was reconstituted with 300 μL methanol/acetonitrile (2:1, v/v), and an aliquot (10 μL) was subjected to UPLC-TOF/MS analysis. The operating conditions for the LC were as follows: column, ACQUITY UPLC^®^ HSS T3 column (1.8 μm particle size, 2.1 × 100 mm, Waters); column temperature, 40 °C; sample room temperature, 4 °C; The mobile phase was water with 10 mM ammonium acetate and 0.1% formic acid (solvent A), and acetonitrile (solvent B); elution program [% of B in A (min)]: 2% (0–3), 2 to 60% (3–10), 60 to 100% (10–20), 100% (20–22), 100 to 2% (22–22.5) and 2% (22.5–25); and flow rate, 0.3 mL/min. Quantitative analyses were carried out in the selected ion monitoring (SIM) mode. Under these conditions, AEA and 2-AG were monitored in their protonated form (m/z 348.2895 (AEA + H) and 396.3117 (2-AG + NH_4_), respectively. As 2-AG undergoes isomerization to 1-AG arising from acyl group migration during sample workup^56^, the peak areas of both isomers were combined for 2-AG quantitation. 2-AG and 1-AG had retention times 15.44 and 15.74 min, respectively.

For the simultaneous quantitation of AEA and 2-AG, a linear regression of the ratio of the peak area counts of AEA and 2-AG to their corresponding internal standards (AEA-*d*_4_ and 2-AG-*d*_8_) versus concentration was used to construct the calibration curves. Calibration curves in clean extraction solvent were used, as the sample matrix always contained 2-AG and AEA. Calibration standard solutions were prepared using stock solutions of AEA-*d*_4_, 2-AG-*d*_8_. To accommodate the two endocannabinoids in the individualized quantitation method, the calibration set consisted of five dilutions, containing AEA-*d*_4_, from 0.01–1 μM (0.01, 0.05, 0.1, 0.5, 1) and containing 2-AG-*d*_8_ from 3.125–50 μM (3.125, 6.25, 12.5, 25, 50). Product ions were monitored in selected ion monitoring (SIM) mode. Injection volumes for samples and standards were 10 μL with needle overfill. Five-point calibration curves were performed in triplicate and 2-AG-*d*_8_ peaks or AEA-*d*_4_ were integrated using Masslynx software version 4.1(Waters Corp., Milford, MA). A linear regression of the ratio of the peak area counts of AEA or 2-AG to their corresponding internal standards (AEA-*d*_4_ and 2-AG-*d*_8_) versus concentration was used to construct the calibration curves. The linear regression correlation coefficients (r^2^) for the AEA and 2-AG calibration curves were 0.999 or higher for all calibration curves.

### RT-PCR

The expression of mRNAs was quantified by real-time RT-PCR as previously described. Primer sequences are shown in Supplemental Table 3. Briefly, total RNA was extracted from hippocampal tissue using the RNeasy Mini Kit (QIAGEN GmbH, Hilden, Germany). The obtained RNA (250 ng) was treated with genomic DNA (gDNA) Eraser for removal gDNA contamination, and reverse-transcribed to cDNA using the PrimeScript RT reagent kit (TaKaRa Bio Inc., Shiga, Japan). The cDNA was amplified with Fast SYBR Green Master Mix (Life Technologies, Carlsbad, CA, U.S.A.), using the StepOnePlus Real-time PCR system (LifeTechnologies). PCR conditions were as follows: 95 °C for 20 s, followed by 40 cycles of 95 °C for 3 s and 60 °C for 30 s. The relative levels of mRNA were determined using the 2^−ΔCT^ method. The amount of target mRNA was normalized to β-actin mRNA, and is shown as a ratio to the control).

### Protein extraction and western blotting

Total protein was extracted from the remaining hippocampal tissue. The hippocampus was homogenized in four volumes (relative to the tissue weight) of 25 mM Tris-HCl (pH 7.4) containing 1 mM EGTA, 1 mM EDTA, 1 mM sodium fluoride, and a protease inhibitor cocktail (Roche Diagnostics, Tokyo, Japan), and centrifuged at 9,000 × *g* for 20 min. Protein concentration was measured by the Lowry assay^57^ . Protein samples (10 µg) were separated on 12 or 15% SDS-polyacrylamide gels and transferred to polyvinylidenedifluoride membrane (PVDF, Millipore, MA) in an ATTO electrophoresis system (ATTO, Tokyo, Japan). After blocking for 1 h at room temperature with TBS-T (Tris-buffered saline with 0.1% Tween) containing 5% skimmed milk and 0.3% BSA, the membranes were at 4 °C overnight with primary antibodies against specific proteins. For the chemiluminescent visualization, membranes were incubated with secondary antibody conjugated with HRP at room temperature for 1 h. After washing six times with TBS containing 0.05% Triton X-100, the membrane was immersed in a Clarity Western ECL substrate (Bio-Rad) for 5 min at room temperature. The relative levels of the target proteins were determined using a ChemiDoc™ MP system operated by Image Lab™ software (Bio-Rad) in the auto-exposure mode.

### Statistical analysis

One-way analysis of variance (ANOVA) was used for performing differences among different groups followed by the Student Newman-Keuls test. For all comparisons, p < 0.05 was considered to indicate statistical significance. The statistical analyses were performed using the Prism software (GraphPad Prism Software, San Diego, CA).

## Supplementary information

Supplemental_information
