# Supplementary material for: Elevation of endocannabinoids in the brain by synthetic cannabinoid JWH-018: mechanism and effect on learning and memory
Source: Sci Rep. 2019 Jul 3;9:9621. doi: 10.1038/s41598-019-45969-4 (PMC6610139; doi:10.1038/s41598-019-45969-4)
Supplement: Supplementary file 1 — Supplemental_information [file 41598_2019_45969_MOESM1_ESM.pdf]

Elevation of endocannabinoids in the brain by synthetic cannabinoid JWH-018: the mechanism and the effect on learning and memory

Ren-shi Li[a,b], Ryo Fukumori[c], Tomoki Takeda[a], Yingxia Song[a], Satoshi Morimoto[d], Ruri Kikura-Hanajiri[e], Taku Yamaguchi[c], Kazuhito Watanabe[f], Kousuke Aritake[f], Yoshitaka Tanaka[g], the late Hideyuki Yamada[a†], Tsuneyuki Yamamoto[c], Yuji Ishii [a*]

[a] *Laboratory of Molecular Life Sciences, Graduate School of Pharmaceutical Sciences, Kyushu University; 3–1–1 Maidashi, Higashi-ku, Fukuoka 812–8582, Japan*

[b]*Research Department of Pharmacognosy, China Pharmaceutical University, Nanjing 211198, People's Republic of China*

[c]*Department of Pharmacotherapeutics and Neuropsychopharmacology, Faculty of Pharmaceutical Sciences, Nagasaki International University, Sasebo, Nagasaki, Japan*

[d]*Division of Pharmacognosy, Graduate School of Pharmaceutical Sciences, Kyushu University, 3-1-1 Maidashi, Higashi-ku, Fukuoka 812-8582, Japan*

[e]*Division of Pharmacognosy, Phytochemistry and Narcotics, National Institute of Health Sciences (NIHS), 3-25-26 Tonomachi, Kawasaki-ku, Kawasaki-city, Kanagawa210-9501, Japan*

[f]*Daiichi University of Pharmacy, 22-1 Tamagawa-cho, Minami-ku Fukuoka 815-8511 Japan*

[g]*Division of Pharmaceutical Cell Biology, Graduate School of Pharmaceutical Sciences, Kyushu University, 3-1-1 Maidashi, Higashi-ku, Fukuoka 812-8582, Japan*

†Deceased

*Corresponding author

Phone: +81-92-642-6586.

FAX: +81-92-642-6588.

E-mail: ishii@phar.kyushu-u.ac.jp

Supplemental Table 1. Suggested components the levels of which were altered by JWH-018 treatment or JWH-018 co-administration of AM251 in the brain of male mice.

	Representative components altered by JWH-018	
sample	Increased	Decreased
Control vs. JWH-018	glutamic acid	*N*-acetyl-aspartate
	2-AG	
	AEA	
	succinic acid	
	acetylcarnitine	
	AMP	
	glutathione disulfide	
	phenylalanine	
	glutamine	
	tyrosine	
	glucose	AEA
	myoinositol	

Supplemental Table 2. Calibration curves of tested endocannabinoids.

Compound	Linear equation	r^2
AEA	y = 8.0879x + 127.4	0.99934
2-AG	y = 19.006x + 18.958	0.99896

Supplemental Table 3. Primer pairs used for real-time RT-PCR for the quantification of mRNA.

Targets	Forward primer (5'->3')<br>Reverse primer (5'->3')	Size of product (bp)	Accession No[a]
FAAH	ttcagaggccttgctggctc<br>acttcccaggcctttcccag	111	NC_000070
phospholipase D	tgttggtgtcctcagcggag<br>ccatgtccctcggcgagaag	165	NC_000071
MAGL	cgcagtagtctggctctagc<br>atcctgcaggggtctttagg	103	NC_000072
DAG	gtgatcagtatccggggaac<br>ccgtaccatgagagtcgact	131	NC_000085
BDNF	tggcctaacaatgtttgcagat<br>ccactcagaaattcctcctgct	155	NC_005102
β-actin	gattactgctctggctccta<br>tcctgcttgctgatccac	135	NM_007393

[a] The GenBank accession numbers are shown.

Abbreviations used are: FAAH, fatty acid amide hydrolase; phospholipase D, *N*-acyl-phosphatidylethanolamines-hydrolyzing; phospholipase D; MAGL, monoacylglycerol lipase; DAG, diacylglycerol and BDNF, brain-derived neurotrophic factor.

Supplementary Figure S1. Full-length blots of Figure 7

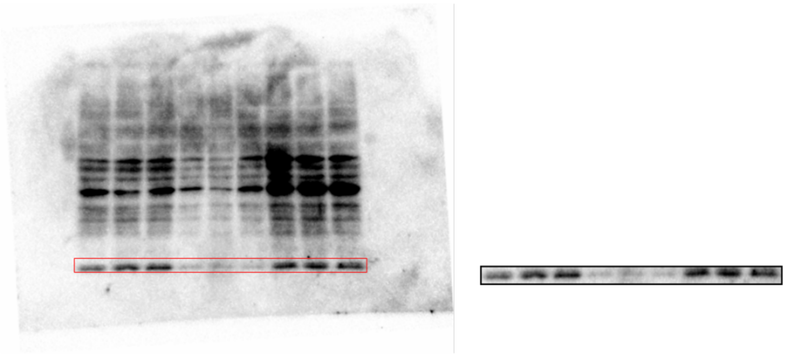

BDNF
